# Successful attentional set-shifting in 2-year-olds with and without Autism Spectrum Disorder

**DOI:** 10.1371/journal.pone.0213903

**Published:** 2019-03-14

**Authors:** Hayley Smith, Alice S. Carter, Erik Blaser, Zsuzsa Kaldy

**Affiliations:** Department of Psychology, University of Massachusetts Boston, Boston, Massachusetts, United States of America; Central European University, HUNGARY

## Abstract

The development of executive function is necessary for flexible and voluntary control of behavior. Deficits in executive function are purported to be a primary cause of behavioral inflexibility—a core clinical symptom—in Autism Spectrum Disorder (ASD). Attentional set-shifting has traditionally been measured with the Dimensional Change Card Sort, however, this task requires following verbal instructions. Here, we used a novel visual search task that does not require verbal instructions in conjunction with eye-tracking to test attentional set-shifting in 2-year-old toddlers diagnosed with ASD (N = 29) and chronological age-matched typically developing controls (N = 30). On each trial, a relevant and an irrelevant target were embedded in a set of feature-conjunction distractors, and toddlers were tasked with searching for the relevant target. Critically, after a set of trials the targets switched roles (i.e., the previously relevant target became irrelevant, and the previously relevant target became irrelevant). We measured visual search performance prior to and following a target switch. We found that both groups of toddlers could readily switch targets, and found strikingly similar performance between typically developing toddlers and toddlers with ASD. Our results challenge the centrality of deficits in attentional set-shifting to early behavioral inflexibility in ASD.

## Introduction

Executive function (EF) is a set of related, higher-order cognitive skills that enable an individual to shift from one mental set to another (set-shifting), suppress a dominant response (inhibitory control), and actively maintain and manipulate mental information (working memory) [1]. EF is considered essential for voluntary and flexible control of thoughts and behavior, and robust EF in childhood is predictive of a range of positive cognitive and socio-emotional outcomes later in life [2]. In typical development, (TD), EF emerges progressively during early childhood, with simpler EF skills emerging prior to more complex ones [3–5]. For example, by the end of the first year of life, infants can successfully maintain a mental representation of a hidden object and inhibit a tendency to search for that object at previous hiding location [6]. During the preschool period, children become increasingly able to flexibly shift attention between conflicting rules. For example, on standard versions of the Dimensional Change Card Sort (DCCS) [7] most children aged 3 show difficulty switching to a new rule that conflicts with the initially learned rule, while most children aged 4–5 switch rules with ease (for a review, see [8]).

There has long been suspicion of a link between EF and behavioral inflexibility in Autism Spectrum Disorder (ASD). Individuals with ASD can get “stuck” on an idea or action, have difficulty with perspective taking, and show distress or oppositional behavior when faced with change (e.g., switching to a new task)–a symptom profile that could readily be explained by a primary deficit in EF; as such, this EF hypothesis has been highly influential in neurocognitive theories of ASD [9–12]. This impairment in behavioral inflexibility maps most naturally onto the set-shifting aspect of EF. And, indeed, there is substantial evidence that set-shifting is impaired in ASD in school-age children and beyond (for reviews, see [13, 14]). Set-shifting tasks involve two phases: (1) an initial training phase where the participant learns a rule (adopts a mental set), and (2) a switch phase, where the participant is tasked with switching to a new rule that conflicts with the initial rule. Set-shifting is expected to incur a ‘switch cost’, i.e., slower response times or increased errors. On classic set-shifting tasks, such as the Wisconsin Card Sort Task [15] individuals with ASD are more likely than TD individuals to continue to implement the initial rule, rather than the new rule (perseverative responding), and/or switch to the new rule then regress to the initial rule (failure to maintain set) [16].

Could differences in set-shifting in EF be a primary deficit in ASD? Investigations into the early development of set-shifting in ASD have been limited. This is surprising given that problems with behavioral flexibility are well-established in young children diagnosed with ASD, and, high levels of repetitive behavior during infancy is predictive of an ASD diagnosis in children at high familial risk [17,18]. Methodological limitations have prevented clear consensus on whether a set-shifting impairment emerges early in ASD. Studies employing simple response shifting paradigms (such as the A-not-B task [19]; or the Spatial Reversal task [20]) suggest deficits in set-shifting are not a primary factor in the development of behavioral inflexibility in ASD: 3-4-year-old children with ASD performed equivalently to chronologically age-matched TD controls [21–23]. On the other hand, studies employing complex attentional set-shifting paradigms such as the DCCS suggest that the development of EF might be delayed: young children with ASD make more perseverative errors and take longer to reach criterion following a switch compared to chronologically age-matched TD controls [24–27]. One potential explanation for this discrepancy is that complex attentional set-shifting tasks like the DCCS impose additional demands on performance that are not present in simple response set-shifting tasks (such as the A-not-B task). For example, attentional set-shifting paradigms often require following complex instructions, fluency with concepts such as shape and color and maintaining a complex rule in mind, and they also impose higher demands on selective attention, and require social interaction [4,28, 29]. What would be ideal is an attentional set-shifting task like the DCCS, but without these additional task demands.

To accomplish this, we used a modified version of our previously validated visual search paradigm [30]. This paradigm was designed for toddlers diagnosed with ASD and age-matched controls, and combines eye-tracking with non-verbal cues that eliminate the need for verbal instructions (TD children vary significantly in their receptive language skills at this age, and toddlers with ASD have known deficits, so a paradigm that eliminates the need for verbal instructions is useful). In this paradigm, we first measured toddlers’ search performance (i.e., the proportion of trials on which they found–fixated–the target) during a baseline phase, then, we switched the identity of the target (a previously irrelevant item became the target: “now the orange carrot is the target, not the green apple”) and performance was again measured. We presented our participants with two of these target switches. Using this approach allowed us to (1) obtain a baseline measure of visual search performance, and (2) quantify the cost to performance, for each of the two target switches.

With regards to the distinction between intra- vs. extra-dimensional shifts in the DCCS (see e. g. [31–33]), what our paradigm requires is more akin to an extra-dimensional shift. Successful performance requires a change in mental set: even though an identical group of 8 items is presented in each of the three phases, participants’ search goal and consequently, gaze behavior, needs to change after each. However, here the two responses–finding the newly-relevant target and finding the now-irrelevant target -–are not mutually exclusive: there is nothing preventing a participant from using extra resources to find the irrelevant target. In our conceptualization, after the target switch, there is a competition between the relevant and irrelevant target. In DCCS, that outcome is winner-take-all, while here both outcomes are measured. Even for a participant or group that shows ‘perfect’ task switching–finding the newly relevant target without cost–it is informative to see how much draw the irrelevant target may have. If ASD is associated with early impairments in set-shifting, then toddlers with ASD should demonstrate a greater switch cost relative to TD controls.

Importantly, our groups of participants were matched on chronological age, not mental age. Thus, toddlers in the ASD group, who had moderate-to-severe clinical symptoms, were also significantly developmentally delayed compared to controls. Non-impaired performance in the ASD group would, therefore, suggest that set-shifting is relatively spared compared to other cognitive skills. Our decision to match groups on chronological age had two motivations: (1) mental age matching would require testing significantly younger TD children, thereby introducing gross differences in their experience [34] and (2) our previous study [30] suggested developmental delay is not a factor in determining baseline visual search performance. This approach also makes for a more sensitive comparison. If there is a deficit associated with ASD diagnosis, it should certainly be evident against chronologically age-matched (as opposed to younger, mental-age matched) controls [24].

## Materials and methods

### Participants

TD children were recruited from the Greater Boston area via mailings. Children diagnosed with ASD were recruited through local early intervention agencies and participation occurred at the beginning of the same visit in which diagnostic testing was later performed. The study protocol and all related materials were approved by University of Massachusetts Boston’s Institutional Review Board (Visual and cognitive processing in typically developing children and children with Autism Spectrum Disorder, Protocol #2008091) and participants were tested in accordance with its ethical guidelines for human research. Written informed consent was obtained from the caregivers of the children who participated in the study. For children included in the ASD group, clinical diagnosis was assigned by a licensed psychologist using the Autism Diagnostic Observation Schedule-2 [35, 36] (Toddler Module, N = 20; Module 1, N = 8; Module 2, N = 1). Calibrated severity scores (CSS) were calculated using the method described by Esler et al. [37]. Children with ASD were also assessed using the Mullen Scales of Early Learning (MSEL; [38]). MSEL testing was not completed for 4 out of 59 participants (3 ASD, 1 TD) because of fussiness or lack of sufficient time. For the TD group, typical development was verified through parental report, the Early Learning Composite standard score (ELC) on the MSEL (an ELC on the MSEL below 70 is 2 SD below the standardized mean and equivalent to a “well below average” level of cognitive development), and scores on the Brief Infant–Toddler Social and Emotional Assessment (BITSEA) [39]. Participants had no known vision impairments or first-degree relatives with colorblindness.

We calculated the minimum required sample size using G*Power 3.1 [40] for a within-between interaction in a repeated-measures ANOVA (effect size = 0.25 (medium), alpha = 0.05). This analysis yielded 44 as the minimum total sample size. The final participant sample, after exclusions, was 59 children (30 children with TD and 29 children diagnosed with ASD). Participants’ chronological age ranged from 15 to 37 months (ASD: *M* = 27.39, *SD* = 4.50; TD: *M* = 27.34, *SD* = 5.80). Participant exclusions were as follows: twenty-four children (13 TD, 11 ASD) were tested and excluded because of poor eye-track quality, 4 children (1 TD; 3 ASD) were excluded because of experimental error, 2 TD children were excluded because of parental interference, and one child with ASD was excluded for failing to attend to the target during the training phase (see Data Analysis). Table 1 shows a summary of demographic information and assessment scores for the final participant sample.

**Table 1:** Summary of participants’ demographic information and scores on standardized assessments.

	ASD Mean (SD)	TD Mean (SD)	*p* / Cohen’s *d*
**N**	29	30	
**# Females**	1	17	
**Age (months)**	27.39 (4.50)	27.34 (5.80)	.972 / .01
**range**	19–37	15–37	
**Mullen Scales:**			
**VR**	35.81 (9.92)	57.62 (10.75)	< .001 / 2.11
**FM**	33.04 (9.97)	48.07 (7.74)	< .001 / 1.68
**RL**	24.15 (10.55)	54.03 (12.50)	< .001 / 2.59
**EL**	29.12 (9.01)	54.86 (10.85)	< .001 / 2.58
**ELC**	64.77 (12.84)	107.41 (15.60)	< .001 / 2.98
**ADOS-2:**			
**SA**	7.97 (1.96)	-	
**RRB**	8.71 (1.19)	-	
**Total**	8.74 (1.44)	-	

T scores (*M* = 50, *SD* = 10) are reported for the following MSEL scales: Visual Reception, (VR), Fine Motor (FM), Receptive Language (RL), and Expressive Language (EL). The Early Learning Composite (ELC) standard score (*M* = 100, *SD* = 15) was computed from these four scales. ADOS-2: Autism Diagnostic Observation Schedule-2, SA: Social Affect, RRB: Restricted, Repetitive Behavior. *P*- and *d*-values are reported for the two-sample *t*-tests comparing the two groups.

### Apparatus

Visual stimuli were displayed on a 17-inch LCD Tobii T120 eye-tracker via Tobii Studio’s presentation software (Tobii Technology, Stockholm, Sweden). Sounds were played through external speakers centered behind the display. Participants and their caregivers were monitored during testing using Tobii Studio’s Live Viewer. Eye movements were recorded at 60 Hz.

### Stimuli

**Fig 1:**
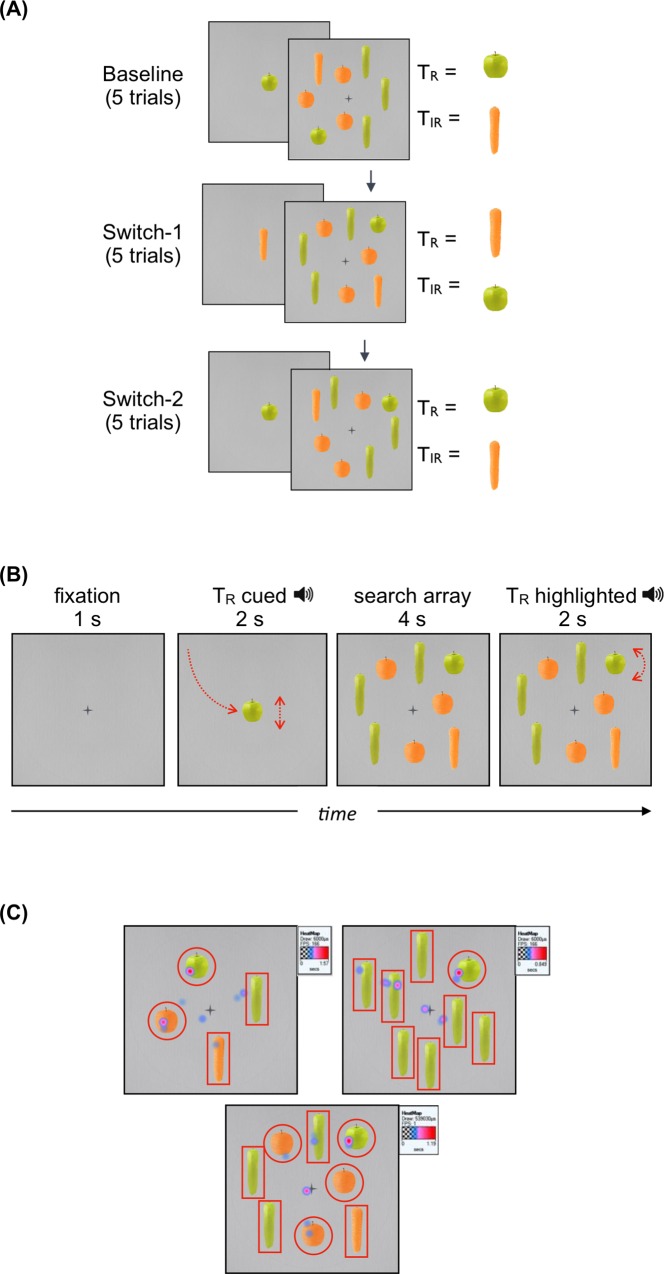
Example stimuli. Panel (A) shows the experimental design. During the Baseline phase, toddlers were tasked with finding the relevant target (T_R_). The identity of T_R_ reversed during the switch phase: the T_R_ became the irrelevant target (T_IR_) and the T_IR_ became the T_R_. Panel (B) shows the event sequence within a trial. Animations are depicted in red. Panel (C) shows the Areas of Interest (AOI) and a heat map of fixations during a Familiarization trial (top left), a Training trial (top right), and a Baseline trial (bottom center) for a TD participant.

Search items were color-rendered photographs of apples (5 × 5°) and carrots (2 × 8°). Search items were presented on a gray background with a 2° fixation cross, and arranged inside a 23° diameter circle (Fig 1). Targets were real-world color/shape combinations (e.g., a green apple and an orange carrot), and all items were nominally isoluminant to each other and the background. Distractors were novel and opposite color/shape combinations (i.e. green carrots and orange apples). To familiarize children with the stimuli, the four types of stimuli were presented twice for 4 seconds prior to the start of the visual search task.

After the two familiarization trials, participants were presented with Training arrays (3 trials): single-feature search displays (color or shape) designed to highlight the special status of the target through ‘pop-out’ [30,41]. Training arrays consisted of the Relevant Target (T_R_) and 8 identical ‘regular’ distractors (e.g., an orange carrot amongst a set of green carrot distractors). Test trial arrays were feature-conjunction displays and consisted of the T_R_ (e.g. an orange carrot), the Irrelevant Target (T_IR_; e.g. a green apple), and 6 ‘regular’ distractors (3 green carrots and 3 orange apples).

To assess attentional set-shifting, we manipulated which of the two targets was ‘relevant’ in each test phase. We did this by using nonverbal cues: The Relevant Target (T_R_) was animated (1) prior to the search display (flying in and ‘honking’) and (2) immediately after the end of the search period (rotating back and forth with a ‘clapping’ sound effect). The Irrelevant Target (T_IR_), meanwhile, remained static.

Test trials were divided into three phases: Baseline, Switch-1, and Switch-2. During the Baseline phase, toddlers were tasked with finding the T_R_, and this was followed by two switch phases (Switch-1 and Switch-2). During the switch phases, toddlers were also tasked with finding the T_R_, yet the identity of T_R_ was reversed: the T_R_ became the T_IR_ and the T_IR_ became the T_R_ (e.g., the orange carrot became irrelevant and the green apple became relevant). Each phase lasted 5 trials and remained in effect until the next target reversal.

The identity of the T_R_ during Baseline was counterbalanced across participants. The quadrant of the T_R_ (upper left, upper right, lower left, lower right) was always next to the quadrant of the T_IR_, and was counterbalanced across trials with the constraint that the T_R_ never occupied the same quadrant as the previous trial. Targets were located approximately equidistant from the center of the screen (Fig 1A).

### Procedure

Participants were seated on their caregiver’s lap, approximately 55–65 cm from the screen. Caregivers wore blacked-out sunglasses or kept their eyes closed, and were instructed not to talk to the child. Participants and their caregivers were monitored during testing to ensure compliance with the experimenter’s instructions. Gaze was calibrated using the standard Tobii infant 5-point procedure (outer corners and center). Once a successful 5-point calibration was achieved, the experiment commenced. At the beginning of each trial, the T_R_ moved into the center of the screen (~1 s) while a cartoon airplane sound effect played. The T_R_ then jiggled (~1 s) while a honking sound effect played. The T_R_ then disappeared, and the search array appeared for 4 s (search period) accompanied by a tick-tock sound. Then, the T_R_ rotated back and forth for ~2 s accompanied by a cartoon applause sound effect (reward animation) (Fig 1B). The entire task lasted approximately 5 min 30 s. Videos containing the entire animation sequence can be downloaded from Open Science Framework (https://osf.io/m8pve/).

### Data analysis

Areas of interest (AOIs) were defined for all search array items (Fig 1C). Gaze positions were averaged between the two eyes to reduce noise. Fixations were defined using the Tobii I-VT filter, which classifies eye movements based on the velocity of the directional shifts of the eye [42].

Participants whose overall track quality was low (i.e., having less than 60% of eye-tracking samples) were excluded from analyses. Data were excluded from trials if (a) looking duration to the entire screen shorter than 100 ms or (2) less than one AOI was fixated. There was no significant difference in the average number of valid trials retained (out of 20) between children with ASD or TD (two-tailed *t*-tests: ASD: *M* = 18.28, *SD* = 1.56; TD: *M* = 18.83, *SD* = 1.46; *t*(57) = -1.418, *p* = .162, *d* = .364) or the average looking to the screen (ASD: *M* = 2791 ms, *SD* = 403 ms; TD: *M* = 2906 ms, *SD* = 384 ms; *t*(57) = 1.126, *p* = .265, *d* = .293).

We computed hit rate (%), average fixation duration (ms), and average fixation latency (ms) for the T_R_ and T_IR_, and for each phase of the experiment. Hit rate was defined as the proportion of trials on which a particular target was fixated. Fixation duration was defined as the average looking time to a particular target. Fixation latency was defined as the average time duration between the first fixation to the screen and the first fixation within a particular target AOI. If a target AOI was not fixated in an entire phase, the value equivalent to the participants’ grand average fixation duration or fixation latency to all AOIs was substituted for the missing value (ASD = 12.32% trials; TD = 12.38% trials).

Classic visual search paradigms measure reaction time in target-present vs. target-absent trials and the efficiency of the search is measured as the slope of the function relating RT and set size [41, 43]. In our paradigm, as the search period was fixed (4 s) and the target was always present, we used hit rates as our main measure of successful search and fixation durations as a measure of task understanding [30].

## Results

In the following analyses, all post hoc tests were Bonferroni-corrected. All data sets met Mauchly’s test for sphericity, so no correction was needed. Data can be downloaded from Open Science Framework (https://osf.io/m8pve/).

### Training phase

#### Hit rates

We included proportion of distractors fixated and average fixation duration to a distractor for reference in the hit rate and fixation duration analyses respectively. To determine whether toddlers looked at the T_R_ more frequently than an average distractor, hit rates were assessed via a 2 (Group: ASD vs. TD) × 2 (Item type: T_R_ vs. Avg. Distractor) mixed ANOVA. Results showed a robust main effect of Item type [*F*(1, 57) = 328.344, *p* < .001, $\eta_{p}^{2}$ = .853]; a main effect of Group [*F*(1,57) = 4.319, *p* = .042, $\eta_{p}^{2}$ = .070]; and no interaction between Group and Item type [*F*(1,57) = .152, *p* = .698, $\eta_{p}^{2}$ = .067]. Post hoc tests showed that the T_R_ hit rate (*M* = 87.53%, *SE* = 2.63%) was higher than the distractor hit rate (*M* = 34.16%, *SE* = 1.30%). The proportion of items visited over the search period was lower for toddlers with ASD (*M* = 57.80%, *SE* = 2.09%) than TD toddlers (*M* = 63.89%, *SE* = 2.05%; *p* = 0.042, *d* = 0.541). Importantly, there was no difference between the two groups in T_R_ hit rate (ASD T_R_: *M* = 85.06%, *SE* = 3.76%; TD T_R_: *M* = 90.00%, *SE* = 3.69%; *p* = 0.35, *d* = 0.245), but hit rates to the Average Distractor were significantly lower in toddlers with ASD (ASD Avg. Distractor: *M* = 30.54%, *SE* = 1.86%; TD Avg. Distractor: *M* = 37.78%, *SE* = 1.83%; *p* = 0.007, *d* = 0.722).

#### Fixation durations

To determine whether toddlers preferentially attended to the T_R_ and successfully followed the nonverbal instructions of our procedure, fixation durations were assessed via a 2 (Group: ASD vs. TD) × 2 (Item type: T_R_ vs. Avg. Distractor) mixed ANOVA. Results showed a significant main effect of Item type [*F*(1, 57) = 39.233, *p* < .001, $\eta_{p}^{2}$ = .408]; no main effect of Group [*F*(1,57) = .196, *p* = .660, $\eta_{p}^{2}$ = .003]; and no interaction between Group and Item type [F(1,57) = .342, *p* = .561, $\eta_{p}^{2}$ = .006]. Post hoc tests showed that on average, the T_R_ was fixated longer (*M* = 829 ms, *SE* = 46 ms) than a distractor (*M* = 524 ms, *SE* = 26 ms). There was no difference in T_R_ fixation duration or the Average Distractor fixation duration between toddlers with ASD and TD toddlers (ASD T_R_: *M* = 802 ms, *SE* = 66 ms; TD T_R_: *M* = 856 ms, *SE* = 65 ms; ASD Avg. Distractor: *M* = 526 ms, *SE* = 37 ms; TD Avg. Distractor: *M* = 522 ms, *SE* = 36 ms).

### Attentional set-shifting

**Fig 2:**
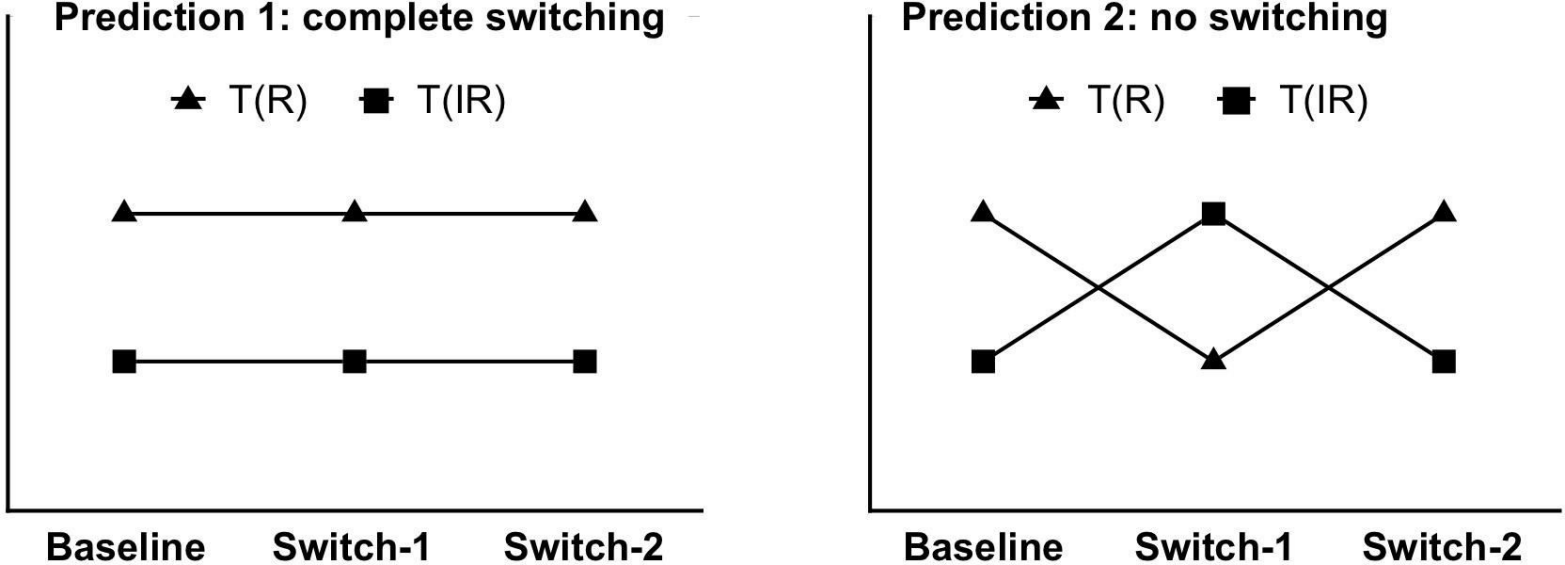
Predicted pattern of results under (A) complete switching and (b) under no switching (complete perseveration) models.

As a reference to interpret the results from the test trials, Fig 2A shows the predictions if participants were able to switch to the new target and away from the old target perfectly, while Fig 2B shows the predictions if no switching occurred (i.e., complete perseveration on the old target).

**Fig 3:**
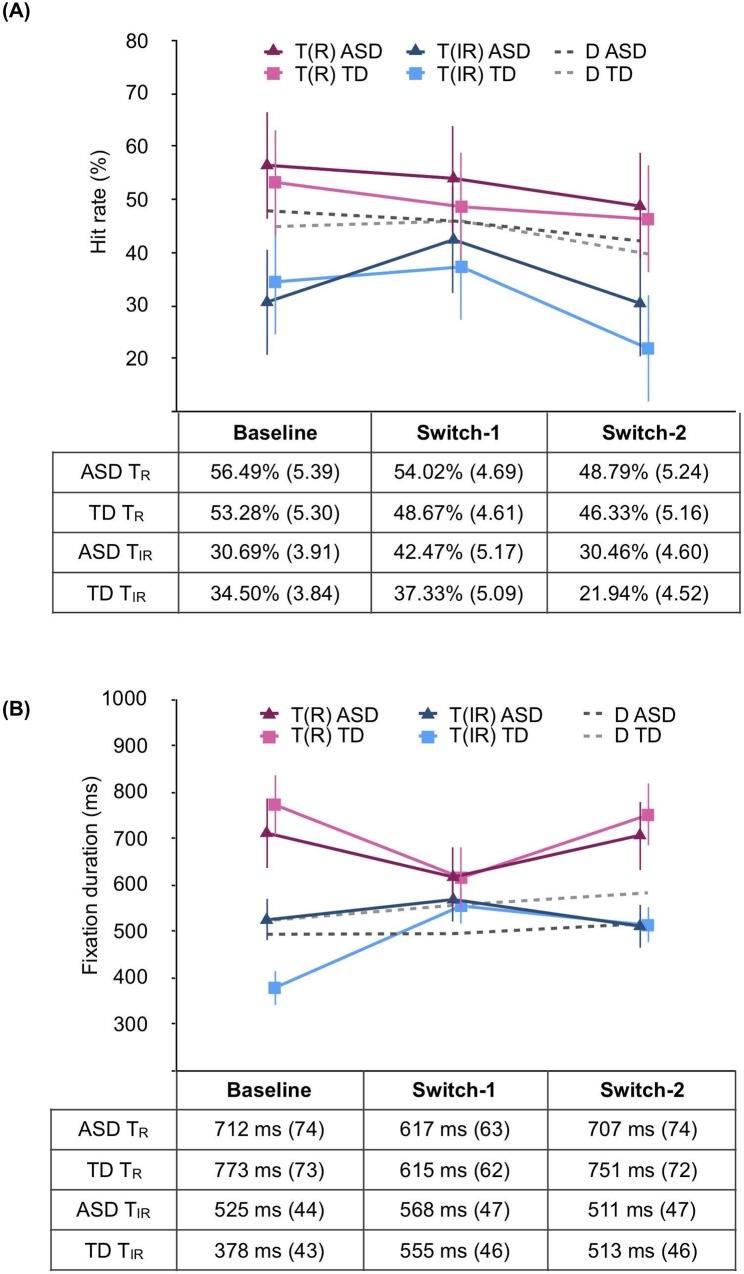
**Hit rates (A) and average fixation durations (B) for the T**_**R**_ **and the T**_**IR**_. Bars are 1 standard error of the mean. Average distractor (D) hit rate and fixation duration is plotted for reference. Mean and SE values are reported below.

**Fig 4:**
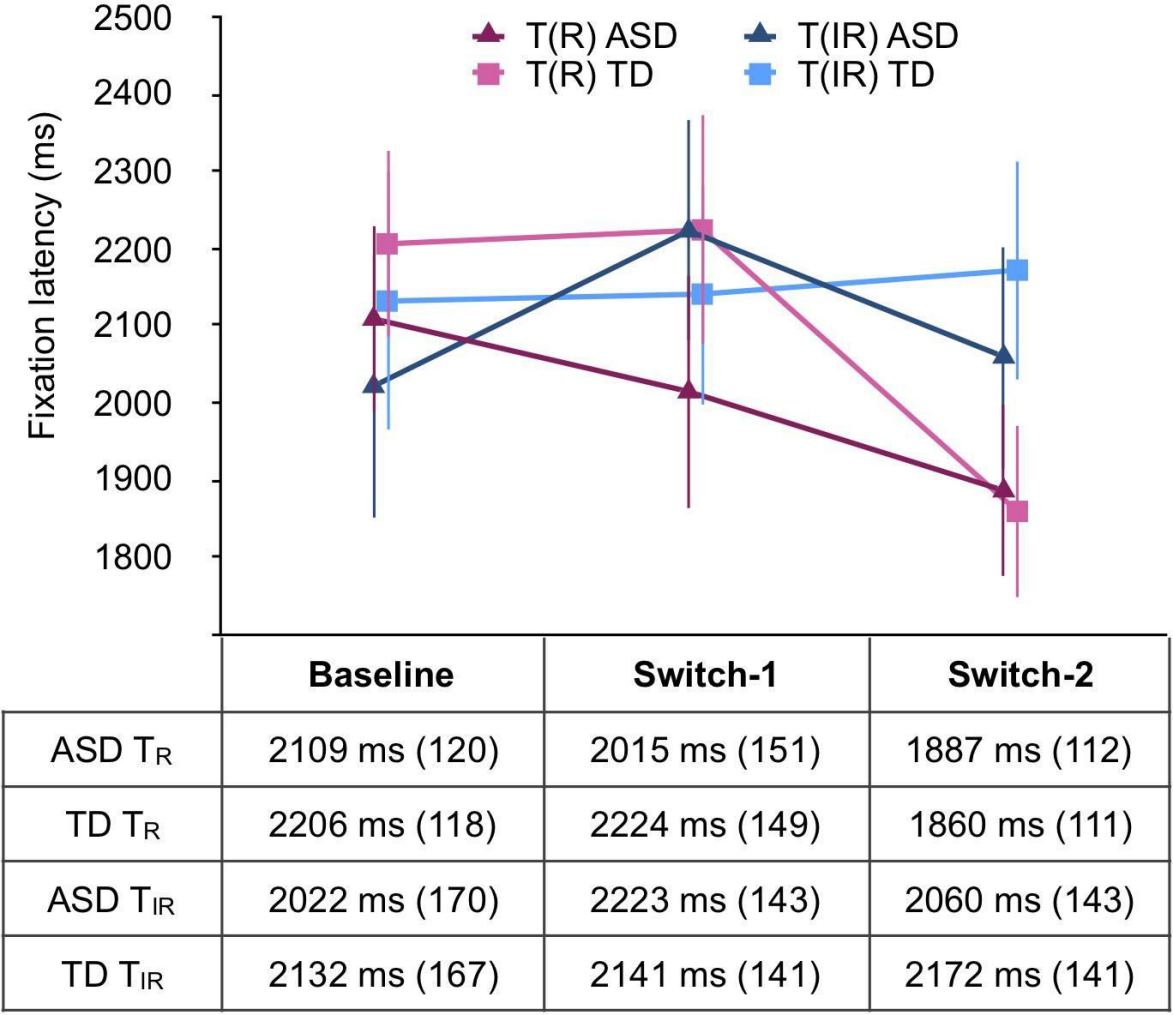
Average fixation latency for the T_R_ and the T_IR_. Bars are 1 standard error of the mean. Mean and SE values are reported below.

For all descriptive data (means, SE) by phase and group, please see Figs 3 and 4.

#### Hit rates

To compare the frequency with which toddlers found the T_R_ and the T_IR_ before and after a target-switch, hit rates were assessed via a 2 (Group: ASD vs. TD) × 2 (Item type: T_R_ vs. T_IR_) x 3 (Phase: Baseline vs. Switch-1 vs. Switch-2) mixed ANOVA. Results showed a significant main effect of Item type [*F*(1, 57) = 41.025, *p* < .001, $\eta_{p}^{2}$ = .419]. The T_R_ hit rate was higher than the T_IR_ hit rate across the three test phases. There was also a significant main effect of Phase [*F*(2, 114) = 4.554, *p* = .013, $\eta_{p}^{2}$ = .074]. Post hoc tests revealed that the (combined) T_R_ and T_IR_ hit rate did not change following the first target switch (i.e., from Baseline to Switch-1) [*p* = 1.0, *d* = .109], however, it did after the second target switch [*p* = .030, *d* = .455]. Importantly, there was no main effect of Group [*F*(1, 57) = 1.1087, *p* = .302, $\eta_{p}^{2}$ = .019]; and no significant interaction effects [all *Fs* ≤ 1.641, *ps* ≥ .198, $\eta_{p}^{2}$ ≤ .028] (Fig 3A).

We found that combined T_R_ and T_IR_ hit rate dropped following the second switch. If this was driven primarily by a drop in T_R_ hit rates, this could indicate that toddlers became less able to execute the task switch, or simply more fatigued. If this was driven by a drop in looking to the T_IR_, this would actually show increased inhibition of the irrelevant target, consistent with successful task switching. We conducted a planned comparison to investigate this. We found that while hit rates to the T_R_ did not change significantly from Switch-1 to Switch-2 [*p* = 1.0, *d* = .138], hit rates to the T_IR_ dropped [*p* = .025, *d* = .647]. Thus, toddlers did not loose interest in finding the relevant target in the third phase, and instead showed an increased ability to suppress their response to the irrelevant target.

#### Fixation durations

We also tested whether toddlers preferentially attended to the T_R_ across the three test phases. Fixation durations were assessed via a 2 (Group: ASD vs. TD) × 2 (Item type: T_R_ vs. T_IR_) x 3 (Phase: Baseline vs. Switch-1 vs. Switch-2) mixed ANOVA. Results showed a robust main effect of Item type [*F*(1, 57) = 23.405, *p* < .001, $\eta_{p}^{2}$ = .291]; and no main effect of Phase [*F*(2, 114) = .331, *p* = .719, $\eta_{p}^{2}$ = .006]; yet a significant interaction between Item type and Phase [*F*(2, 114) = 5.109, *p* = .008, $\eta_{p}^{2}$ = .082]. Again, there was no main effect of Group [*F*(1, 57) = .147, *p* = .807, $\eta_{p}^{2}$ = .001]. No other interaction effects were significant [all *Fs* ≤ 1.266, *ps* ≥ .265, $\eta_{p}^{2}$ ≤ .022] (Fig 3B).

Post hoc tests were conducted to further explore the Item type and Phase interaction. To determine whether children preferentially attended to the T_R_, we compared fixation duration to the T_R_ and T_IR_, separately for each phase. During Baseline, children fixated the T_R_ longer than the T_IR_ [*p* < .001, *d* = .884]. However, during Switch-1, there was no significant difference between fixation duration to either target [*p* = .351, *d* = .184]. During Switch-2, children again fixated the T_R_ longer than the T_IR_ [*p* = .001, *d* = .660].

To determine whether the target-switch resulted in fixation duration cost, we compared fixation durations to the T_R_ and T_IR_ across Phases. For the T_R_, fixation duration was not significantly different at Baseline compared to Switch-1 [*p* = .153, *d* = .342], or at Switch-2 compared to Switch-1 [*p* = .193, *d* = .308]. By contrast, both groups of children fixated the T_IR_ longer following the first target switch (i.e., Switch-1) relative to the Baseline phase [*p* = .035, *d* = .448]. There was no significant change in fixation duration between Switch-1 and Switch-2 for the T_IR_ [*p* = .828, *d* = .198].

#### Fixation latencies

Finally, we performed a 2 (Group: ASD vs. TD) × 2 (Item type: T_R_ vs. T_IR_) x 3 (Phase: Baseline vs. Switch-1 vs. Switch-2) mixed ANOVA on fixation latencies. There was no significant main effect of Target type [*F*(1, 57) = .978, *p* = .327, $\eta_{p}^{2}$ = .017], Phase [*F*(2, 114) = 1.596, *p* = .207, $\eta_{p}^{2}$ = .027], or Group [*F*(1, 57) = .548, *p* = .462, $\eta_{p}^{2}$ = .010]. None of the interaction effects were significant either [all *F*s ≤ 1.308, *p*s ≥ .274, $\eta_{p}^{2}$ ≤ .022] (Fig 4).

### Relations with participant characteristics

Our main dependent measure of success is how often children could find the target (T_R_ hit rate); whether they could change their search behavior (find the newly relevant target) with changing task demands. Our secondary measure is whether they appreciated the target’s special status, once they found it (T_R_ fixation duration). Even though we did not find any significant differences in T_R_ hit rates across phases and between the groups, it is possible that individual differences are related to certain participant characteristics. For our dependent variables, we computed each participant’s switch cost with respect to the T_R_ at Baseline for both hit rate and fixation duration and for each of the target switches separately. We did this by subtracting performance at Baseline from Switch-1 performance (Switch-1 cost), and performance at Baseline from Switch-2 performance (Switch-2 cost).

We conducted multiple linear regressions for each of our four dependent variables (with hit rate switch costs being more central to task performance). We entered the following predictors in our model: Group, Chronological Age (in days), Mental age (Mullen ELC), the interaction of Mental Age and Group, and the interaction of Mental age and Chronological Age. All variables were centered. None of the overall regression equations were significant [all *F*s(5, 49) ≤ 1.819, *p* ≥ .126]. However, when examining individual predictors, for Hit rate Switch-2 cost, Mental age was a significant predictor [*p* < .024]. The effect of the Mental age*Group interaction term was not significant [*p* < .221]. Children (independent of diagnostic status) with lower mental age had higher hit rate switch costs following the second target switch. For a summary of coefficients in the model, please see Table 2.

**Table 2:** Summary for model fit (predicting Hit rate change following the second target switch). * *p* < .05.

	B	SE	β	*t*	*p*
Group	41.408	31.216	.328	1.326	.191
Mental age	.895	.383	.659	2.335	.024*
Age	-.007	.030	-.032	-.237	.814
Mental age*Age	-.003	.001	-.256	-1.853	.070
Mental age*Group	-1.553	1.254	-.301	-1.239	.221

We also tested whether ASD symptom severity (ADOS-2 Total CSS) in the ASD group predicted any of our switch cost measures using linear regressions, but none of those effects were significant [all *F*s(1, 27) ≤ .310, *p*s ≥ .582].

## Discussion

In this study we employed a novel, nonverbal eye-tracking visual search paradigm to test attentional set-shifting in 2-year-old toddlers with and without a diagnosis of ASD. Contrary to the findings from studies with preschool-age children using attentional set-shifting tasks (e.g. [24,26]), we found no evidence of impaired attentional set-shifting in young toddlers with ASD.

In our experiment, participants were tasked with searching for one of two potentially relevant targets, with which target was relevant indicated by non-verbal cues. The crucial manipulation in our study was that after searching for a particular target during a baseline phase, the two targets switched roles, with the previously irrelevant target becoming the relevant target and vice versa. At Baseline, both groups of toddlers found the relevant target more often, and fixated it longer, than the irrelevant target. Importantly, following the first target switch, both groups of toddlers found the (newly) relevant target more often than the (now, demoted) irrelevant target. Fixation durations no longer differed significantly between the two targets after the first switch (while fixation duration to the relevant target was equal in the first two phases, the now irrelevant target was fixated longer after the switch). The first finding shows that toddlers updated the identity of the target–successfully switched tasks—while the second shows that the prior role of the now irrelevant target was still appreciated. This effect of selection history has been observed in visual search studies with adults [44]. Following the second target switch, both groups of toddlers still found the relevant target more often (and again fixated it longer) than the now irrelevant target. In short, both groups were able to task-switch, and the performance of toddlers with ASD did not differ from age-matched TD controls.

We think it is important to provide evidence here that task execution in this paradigm was goal-driven and intentional–that finding the target on test trials was not driven by automatic, low-level visual orienting. The evidence directly from our study stems from a comparison of the hit rates in the single-feature training trials (by design, feed-forward, ‘efficient’ search) to the feature-conjunction test trials (assumedly ‘guided’, relatively inefficient, search). Target hit rates in the training trials were much higher (87.0%) than in the test trials (Baseline phase, 54.9%). This wide gap gives us assurance that these two tasks were indeed fundamentally different; that there was nothing about the procedure or parameters of the test trials that inadvertently rendered search bottom-up or automatic, as it is in training trials. And, in an earlier published study from our lab [30], with a nearly identical paradigm and populations (18-36-month-old TD toddlers and toddlers diagnosed with ASD), we had found robust set size effects; the classic signature of guided search. To make the case that search here had a top-down component, we can look to another study from our lab [45] (again with nearly identical paradigm and populations), where we found that an individual’s search performance was modulated by his/her cognitive effort, as indexed by pupil dilation during search.

The lack of a performance difference between our groups is striking, given that the toddlers with ASD in our sample, as is typical, had significantly lower mental age than our age-matched controls (Mullen ELC: ASD: *M* = 64.77, *SD* = 12.84; TD: *M* = 107.41, *SD* = 15.60, *p* < .001, *d* = 2.984) and had moderate-to-severe ASD symptoms. If a deficit in attentional set-shifting is associated with ASD diagnosis, it should have been evident against chronologically age-matched (as opposed to younger, mental-age matched) controls [24].

We also explored whether attentional set-shifting performance was related to participant characteristics, such as chronological age, mental age, or diagnostic status. None of these characteristics predicted our main measures of performance, target hit rate changes across phases, except for mental age, and there only following the second target switch. Children with lower mental age showed a higher switch cost (independent of diagnostic status). While this is consistent with a model of developing EF skills, future studies are needed to corroborate this further. The fact that symptom severity (ADOS-2 Total CSS) was not related to any of our experimental measures in the ASD group is not surprising, since we did not find any group differences; performance was not related to diagnostic status.

Behavioral inflexibility is a central feature of ASD and is a major barrier to social interaction and learning. To date, investigations of set-shifting in children with ASD have yielded inconsistent findings. Studies employing complex attentional set-shifting paradigms such as the DCCS have suggested that the development of this ability is already delayed in early childhood, whereas studies with simple response set-shifting have suggested deficits are later emerging, and thus secondary to the development of core symptoms of ASD. As our task was a feature-conjunction search, to perform well, toddlers needed to maintain an attentional set that included the relevant target features (e.g., green/apple), and update it following a target switch (e.g., orange/carrot). Toddlers were not “instructed” to ignore the previously relevant but now irrelevant target. Thus, we can conclude that when the verbal and inhibitory control demands of a task are low, young toddlers with ASD do not exhibit impairment in attentional set-shifting.

It is remarkable that toddlers with ASD performed as well as the age-matched TD controls in our task, given the substantial difference in cognitive developmental level. That said, we did not see a significant ‘ASD advantage’ in visual search as expected from prior work (reviewed in [46] and in contrast to our findings with a similar paradigm [30]). There are two potential explanations for this difference. First, in our prior study using a similar paradigm [30], the group difference emerged after a longer series of trials and, in fact, a visual search advantage was not present during the equivalent period (i.e., the first 5 trials) in that study. Second, to ensure that the search array items were equally perceptually salient, in the current study, we rendered all items nominally isoluminant to each other and the background (thus, only hue differences remained between the items). Attention to color (hue) differences has been shown to be impaired in children with ASD [47,48], so this may have affected the search of our toddlers diagnosed with ASD more so than TD toddlers.

How might we reconcile the findings from the current study with our knowledge of the development of brain mechanisms underlying attentional set-shifting? In the typical adult brain, successful set-shifting is dependent on a fronto-parietal central executive network, which consists of the ventrolateral prefrontal cortex, inferior frontal cortex, posterior parietal cortex, anterior insula, and the anterior cingulate cortex [49–51]. In young TD children, fNIRS studies have found that differences in the activation of the prefrontal cortex [52] and interactions between frontal and posterior parietal regions [53] mediate successful set-shifting. While the neural mechanisms of attentional set-shifting in young children with and without ASD has yet to be contrasted, recent evidence suggests atypical functional connectivity of the dorsal and ventral attention networks from infancy to adolescence in ASD [53–56]. Selective attention is essential for successful set-shifting [57] and future research should examine how the early development of this system affects EF in ASD [50, 58].

Our findings extend the developmental EF literature in two important ways. First, we introduced a novel eye-tracking task to measure attentional set-shifting performance that can be used with children under 3 years of age. To date, the youngest age at which attentional set-shifting has been studied in TD toddlers, using verbal instructions, was at 30 months [59, 60]. Our task does not require verbal instructions or response, reducing the effect of verbal fluency on performance, and making it ideal for studying young or atypically developing populations. Furthermore, our paradigm is based on gaze behavior. Previous research has shown that eye-tracking is a sensitive measure of attentional differences between children with and without ASD [61, 62]. To date, the use of eye-tracking to study attentional set-shifting has been limited to older TD children and adults [63, 64]. Second, in this novel paradigm with low task demands, both TD children and children with ASD showed successful attentional set-shifting at 2 years of age. Attentional set-shifting is necessary for many higher-order cognitive tasks, and our study represents the first step in studying it in young toddlers with typical and atypical development. Our findings suggest that despite clear problems with behavioral flexibility, deficits in attentional set-shifting may not be a central feature of ASD at the earliest age the condition can be diagnosed.
